# Association of serum transaminases with short- and long-term outcomes in patients with ST-elevation myocardial infarction undergoing primary percutaneous coronary intervention

**DOI:** 10.1186/s12872-017-0485-6

**Published:** 2017-01-28

**Authors:** Ming Gao, Yi Cheng, Yang Zheng, Weihua Zhang, Lin Wang, Ling Qin

**Affiliations:** 1 The Cardiovascular Center, the First Hospital of Jilin University, 71 Xinmin Street, Changchun, 130021 China; 2 Laboratory for Cardiovascular Diseases, Institute of Translational Medicine, the First Hospital of Jilin University, Changchun, China; 3 Key Laboratory for Cardiovascular Mechanism of Traditional Chinese Medicine, the First Hospital of Jilin University, Changchun, China

**Keywords:** ST-elevation myocardial infarction, Serum transaminase, Primary percutaneous coronary intervention

## Abstract

**Background:**

Alanine transaminase (ALT) and aspartate aminotransferase (AST) are referred to as liver transaminases. Although used routinely in clinical practice for decades, their role as predictors of mortality has not been examined until recently. We studied the predictive value of these serum transaminases in patients with ST-segment elevation myocardial infarction (STEMI) treated with primary percutaneous coronary intervention (PCI).

**Methods:**

We analyzed records of 2417 consecutive STEMI patients with no preexisting liver disease who were treated with primary PCI at the Cardiovascular Center in the First Hospital of Jilin University. The outcomes measured were all-cause mortality at the first month and at 2 years. The relationship between the baseline serum transaminase levels and primary outcome was determined.

**Results:**

We found a significant correlation between elevated liver transaminases and the Killip classification (*P* < 0.001 for ALT; *P* < 0.001 for AST), cardiac troponin I (*P* = 0.002 for ALT; *P* < 0.001 for AST), infarct-related coronary artery (*P* = 0.036 for ALT; *P* = 0.011 for AST), and pre-thrombolysis-in-myocardial-infarction (pre-TIMI) flow (*P* < 0.001 for ALT and AST). The serum level of ALT and AST were high along with the increasing of the grade of Killip classification. The primary infarct-related coronary artery in patients with ALT ≥95th percentage was left anterior descending artery (56%), followed by right coronary artery (36%). The OR for all-cause mortality at 2 years for participants with ALT ≥95th percentage was 5.370 (95% CI: 2.899–9.948), 7.034 (95% CI: 3.718–13.307) after adjustment for age and gender and 1.051 (95% CI: 0.302–3.652) after adjustment for all covariables. The OR for all-cause mortality at 2 years for participants with AST ≥95th percentage was 5.370 (95% CI 2.899–9.948) and 5.699 (95% CI 3.030–10.718) after adjustment for age and gender and 1.796 (95% CI: 0.588–5.481) after adjustment for all covariables. ALT (HR 1.004, 95% CI 1.001–1.006, *P* = 0.010) and AST (HR 0.999, 95% CI 0.998–1.000, *P* = 0.030) were associated with early all-cause mortality in patients with STEMI treated with PCI but not at 2 years post-procedure, unless for AST and ALT levels ≥95th percentage. Moreover, short- and long-term outcomes were significantly worse when both AST and ALT levels ≥95th percentage (*P* < 0.001).

**Conclusions:**

Serum transaminases ≥95th percentage were associated with a significantly increased incidence of short- and long-term all-cause mortality.

**Trial registration:**

Registration number: ChiCTR-EPC-16008199, 31 March 2016.

**Electronic supplementary material:**

The online version of this article (doi:10.1186/s12872-017-0485-6) contains supplementary material, which is available to authorized users.

## Background

Alanine transaminase (ALT) and aspartate aminotransferase (AST) are referred to as liver transaminases. Although their levels have been tested routinely in clinical practice for decades, their role as predictors of mortality has not been examined until recently. ALT is found predominantly in hepatocytes and is a widely used specific serum marker of liver disease. AST is mainly derived from the liver; although, a significant portion is derived from other tissues, such as heart, red blood cells, and muscle, which makes it an imperfect marker of liver function. Recent studies have demonstrated an increasing interest in investigating the role of liver transaminases in independently predicting cardiac-related morbidity and mortality [1–3].

Several prospective epidemiological studies have suggested that hepatic dysfunction is common in cardiac disease [4, 5]. If no other causes of liver injury are identified, the elevations of liver aminotransferases are associated with a higher incidence of cardiac-related mortality [6]. However, the results from these studies have been inconsistent and have revealed geographical variations in the association between ALT and all-cause mortality [7–11]. Little is known about the association between hepatic dysfunction and mortality in patients with ST-segment elevation myocardial infarction (STEMI) treated with stents or angioplasty for coronary artery stenosis. The goal of this study was to evaluate the association between elevated liver transaminases and all-cause mortality in patients with STEMI undergoing primary percutaneous coronary intervention (PCI) at 1-month and 2-years post-procedure.

## Methods

### Study population

In this study, we examined digital data from the First Hospital of Jilin University, Cardiovascular Department, which is located in an agricultural province of Northeast China, populated by members of the Chinese Han ethnic group. This prospective observational cohort study aimed to investigate the association between ALT and AST levels and the incidence of all-cause mortality at 1 month and 2 years following treatment. From January 1, 2013 to December 31, 2014, 6017 patients were hospitalized with a diagnosis of acute myocardial infarction. Among those patients, 3692 had ST-segment elevation myocardial infarction. From those patients, 1056 were excluded who did not undergo angiography. Finally we included in this study all 2636 (71.3%) consecutive STEMI patients who underwent PCI without thrombolysis or conservative therapy. From this group, we excluded 219 patients for the following reasons: insufficient AST or ALT data (*n* = 126), cirrhosis (*n* = 1), fatty liver (*n* = 64), and hepatitis (*n* = 8). In addition, we excluded 14 patients of another ethnic group. Finally, we excluded 16 patients because there was missing follow-up information. The final number of patients enrolled in this study was 2417. Their baseline demographic data, medical history, laboratory data, and clinical data during hospitalization were retrieved from the department’s heart disease electronic database (Table 1). The mean time to follow up was 2 years (30–1165 days) in the included patients. Angiographic features, angiographic results, and treatment characteristics are provided in Table 2. The study protocol was approved by the ethics review board of the First Hospital of Jilin University (No.2016-263).

**Table 1:** Baseline characteristics of participants (*n* = 2417)

Characteristic	N (%) or mean ± SD
Demographic data	
Age (year)	59.5 ± 11.1
Male, n (%)	1748 (72)
Living in the city	11,148 (48)
Hospital days	7.2 ± 3.8
Medical history (n)	
Dyslipidemia	931 (38)
Diabetes mellitus	545 (23)
Hypertension	1088 (45)
Myocardial disease	160 (7)
Previous myocardial infarction	24 (1)
Arrhythmias	492 (20)
Previous stroke	75 (3)
Malignancies	6 (0.2)
Peripheral vascular disease	24 (1)
COPD	30 (1)
Infarct location by ECG	
Inferior	1189 (49)
Anterior	1172 (48)
Lateral	56 (2)
Killip classification	
Cardiogenic shock	101 (4)
Noncardiogenic shock	2316 (96)
Laboratory data	
K^+^ (mmol/L)	3.9 ± 0.5
Creatinine (mmol/L)	75.3 ± 29.8
Na^+^ (mmol/L)	139.4 ± 4.1
Total cholesterol (mmol/L)	4.5 ± 1.0
HDL-C (mmol/L)	1.1 ± 0.3
LDL-C (mmol/L)	2.8 ± 0.9
Triglycerides (mmol/L)	1.7 ± 1.2
Glucose (mmol/L)	7.1 ± 3.1
ALT (unit/L)	55.5 ± 108.3
AST (unit/L)	165.3 ± 283.1
ALP (unit/L)	72.4 ± 24.4
GGT (unit/L)	47.4 ± 69.4
Cardiac troponin I (ng/mL)	20.9 ± 60.9
Creatine kinase MB (ng/ml)	38.3 ± 75.9
NT-proBNP (pg/mL)	1829.9 ± 6019.6

*SD* standard deviation, *COPD* chronic obstructive pulmonary disease, *ECG* electrocardiographic, *HDL-C* high-density lipoprotein cholesterol, *LDL-C* low-density lipoprotein cholesterol, *ALT* alanine aminotransferase, *AST* aspartate aminotransferase, *ALP* Alkaline phosphatase, *GGT* ɤ-glutamyl transpeptidase, *NT-proBNP* N-terminal pro-brain natriuretic peptide

**Table 2:** Angiographic features, angioplasty results, and mortality of participants (*n* = 2417)

Characteristic/Outcome	N (%)
Infarct related artery	
RCA	1044 (43)
LAD	1,172 (48)
LCX	201 (8)
Pre-TMI flow	
TIMI-0 flow	475 (20)
≥ TIMI −1 flow	1,942 (80)
Multivessel disease (≥2 vessels)	1145 (47)
Method of reperfusion, n (%)	
Balloon	282 (12)
Stent implantation	2135 (88)
All-cause mortality	
1-month death	59 (2)
24-month death	128 (5)

*RCA* right coronary artery, *LAD* left anterior descending, *LCX* left circumflex coronary artery, *TIMI* thrombolysis in myocardial infarction

### Laboratory data

Peripheral blood samples were obtained at the time of admission and the following were tested: creatinine, cardiac troponin I (Tn I), creatine kinase MB (CKMB), K^+^, Na^+^, and N-terminal pro-brain natriuretic peptide (NT-proBNP). Total cholesterol (TC), triglyceride (TG), high-density lipoprotein cholesterol (HDL-C), glucose, ALT, AST, alkaline phosphatase (ALP), ɤ-glutamyl transpeptidase (GGT), and low-density lipoprotein cholesterol (LDL-C) were measured after 12-hours fasting following admission. All blood samples obtained at the time of admission were analyzed in the certified laboratory department of First Hospital of Jilin University. ALT and AST were determined using ultraviolet-lactate dehydrogenase method and ultraviolet-malic acid dehydrogenase method according to the manufacturers’ instructions (Kehua Bio-Engineering, China). According to local recommended guidelines, an abnormal ALT level was defined as a serum concentration >50 U/L for men and >40 U/L for women; an abnormal AST level was defined as a serum concentration >40 U/L for men and >35 U/L for women.

### Protocol and definition

STEMI was diagnosed in accordance with the European Society of Cardiology/American College of Cardiology consensus document, using at least two separate measurements for the following parameters: a past history of chest pain, diagnostic electrocardiographic changes, and serial elevation of serum cardiac biomarkers [12]. The Killip classification is a system used in individuals with an acute myocardial infarction, taking physical examination and the development of heart failure to predict and stratify their risk [13]. It was determined as follows: Killip class I, no clinical signs of heart failure; Killip class II, rales or crackles in the lungs, an S_3_, and elevated jugular venous pressure; Killip class III, frank acute pulmonary edema; and Killip class IV, cardiogenic shock or hypotension and evidence of peripheral vasoconstriction [13].

All patients enrolled were pre-medicated with either 1) 300 mg aspirin or 2) 600 mg clopidogrel orally and unfractionated heparin (1000 IU/L intravenous injection). Coronary angiography was performed through the femoral or radial artery using standard techniques to evaluate coronary artery lesions. Multivessel coronary artery disease was defined as significant stenosis of two or more major coronary arteries, stenosis of ≥50% of the diameter in the left main or proximal segment of the left anterior descending artery (LAD), and stenosis of ≥70% of the diameter in two of these three vessels: left coronary artery, right coronary artery (RCA), and left circumflex coronary artery (LCX). An artery was considered infarct-related if one of the following criteria was present: definite or suspected thrombus, a ruptured or ulcerated plaque, the presence of thrombolysis-in-myocardial-infarction (TIMI) flow grade ≤2, or stenosis ≥70% of the diameter. The coronary artery flow was assessed using TIMI flow grades [14]. The strategy for multivessel arteries disease in our center was infarct-related artery only according to current guideline recommendations which encourage culprit-only PCI in patients with STEMI and multivessel disease (excluding cardiogenic shock) [15, 16].

### Follow-up

To monitor for all-cause mortality following discharge, the clinical course of the patients was monitored by telephone until death or April 1, 2016, whichever occurred first. In the case of patients who were lost to follow-up, we reviewed all medical records and contacted the patients’ families. The endpoint of the present study was defined in separate analyses of all-cause mortality at 1-month and at 2-years post-procedure. Two independent observers performed statistical analysis.

### Statistical analysis

After testing for normality by the Kolmogorov-Smirnov test, the data were presented as mean ± standard deviation (SD) or median [interquartile range]. Separate analyses were conducted for ALT and AST. Serum ALT and AST levels were categorized into percentiles separately (<25th, 25th to <50th, 50th to <75th, 75th to <95th, and ≥95th percentages). Liver enzyme levels <25th percentage were used as a reference. Logistic regression was performed to evaluate the power of percentiles for each liver enzyme with all-cause mortality. AUC curves were constructed to further illuminate the best cut-off values for ALT and AST for predicting the all-cause mortality. Multiple linear regression was used in order to test the association of the liver enzymes and other previously identified prognostic factors in STEMI with the measured outcomes. Survival curves were generated by the Kaplan–Meier method, and survival among groups was compared using the Log Rank test. To evaluate the independent contribution of the baseline clinical characteristics, medical history, and laboratory data on the occurrence of major events, two consecutive models of Cox multiple regression analysis were performed with forward stepwise selection to determine predictors for all-cause mortality at 1 month and 2 years separately. In the first model, the baseline clinical characteristics and laboratory data were included. Only those clinical and laboratory variables found to be independent in the first model were included in the second model. For all analyses, a two-sided *P* < 0.05 was considered statistically significant. All analyses were conducted using Stata software, version 12 (Stata Corp., College Station, TX).

## Results

### Baseline characteristics

The baseline data of the participants included demographic characteristics, medical history, laboratory data, culprit vessel, and PCI procedure. The median age of the 2417 patients was 60 years [interquartile range: 52–67] with 72% male patients. Using the definitions of abnormal liver enzyme levels defined previously, 38.9% of the study population had elevated ALT and 71.9% of the study population had elevated AST (Table 1). During the median 2 years (ranging from 30 to 1165 days, median 775 days) of follow up, the cumulative data for all-cause mortality was 2% (59 deaths) at the first month and 5% (122 deaths) at 2 years (Table 2).

### Risk factors and elevated liver enzymes

**Fig. 1:**
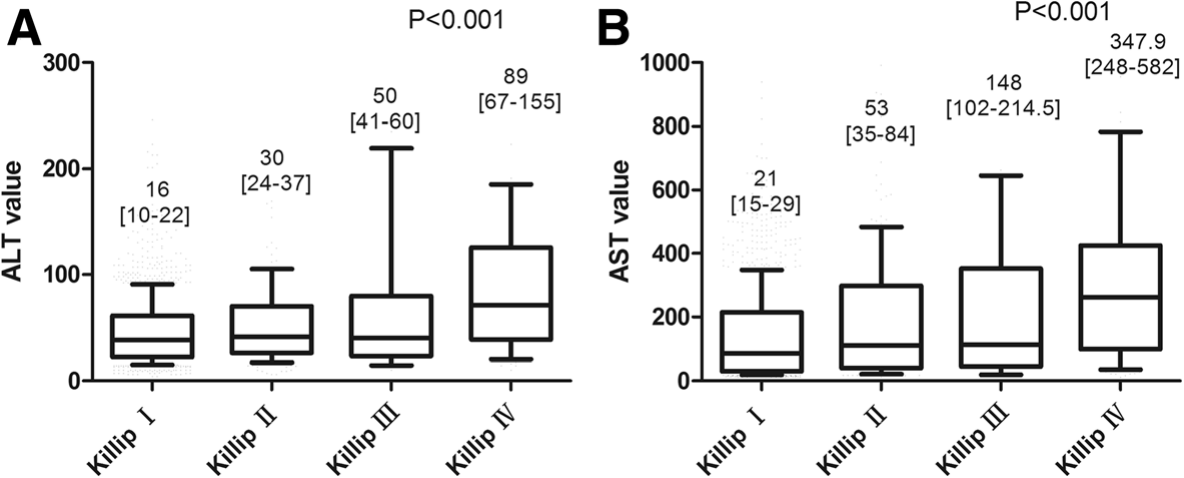
ALT (**a**) and AST (**b**) values stratified according to Killip classification. The *horizontal line* shows the median value. The box showed the interquartile range. The *vertical line* shows the 10th–90th percentage

We found some differences in the associations between known risk factors and elevated liver enzymes between ALT and AST. Variables found to be independent in the first regression model and further analyzed included age, gender, living in rural regions, hospital days, medical history, Killip classification, infarct-related coronary artery, Tn I, pre-TIMI, and multivessel disease. In multivariate regression analysis, elevated ALT and AST were both related to Killip classification (*P* < 0.001 for ALT; *P* < 0.001 for AST), Tn I (*P* = 0.002 for ALT; *P* < 0.001 for AST), infarct-related coronary artery (*P* = 0.036 for ALT; *P* = 0.011 for AST), and pre-TIMI flow (*P* < 0.001 for ALT and AST). ALT was also related to age (*P* = 0.002) and dyslipidemia (*P* = 0.048) (Table 3). The serum level of ALT and AST were high along with the increasing of the grade of Killip classification (Fig. 1). The primary infarct-related coronary artery in patients with ALT ≥95th percentage was LAD (56%), followed by RCA (36%).

**Table 3:** Association between liver enzymes and covariables in univariate analyses

Variable	CC for ALT (SE)	*P* value	CC for AST (SE)	*P* value
Age (year)	30.456 (3.275)	0.002	−0.467 (0.546)	0.392
Male, n (%)	4.316 (5.199)	0.407	14.206 (13.344)	0.287
Living in the city	−5.163 (4.548)	0.256	−17.423 (11.674)	0.136
Hospital days	0.164 (0.602)	0.785	−2.807 (1.545)	0..069
Dyslipidemia (n)	−9.094 (4.604)	0.048	−20.057 (11.818)	0.090
Diabetes mellitus (n)	−3.187 (5.399)	0.555	−11.918 (13.859)	0.390
Hypertension (n)	1.631 (4.536)	0.719	3.295 (11.642)	0.777
Myocardial disease (n)	−17.047 (9.239)	0.065	−38.681 (23.715)	0.103
Killip classification	30.456 (3.275)	<0.001	83.942 (8.406)	<0.001
Infarct-related coronary artery	8.766 (4.176)	0.036	27.303 (10.718)	0.011
cardiac troponin I (ng/mL)	0.116 (0.037)	0.002	0.809 (0.094)	<0.001
Pre-TMI flow	−29.730 (5.587)	<0.001	−69.304 (14.339)	<0.001
Multivessel disease (≥2 vessels)	7.055 (4.539)	0.120	17.559 (11.650)	0.132

*CC* correlation coefficient, *ALT* alanine aminotransferase, *SE* standard error, *AST* aspartate aminotransferase, *NT-proBNP* N-terminal pro-brain natriuretic peptide

At cox proportional hazards regression, ALT (hazard ratio [HR] 1.004, 95% confidence interval [CI] 1.001–1.007, *P* = 0.010) and AST (HR 0.999, 95% CI 0.098–1.000, *P* = 0.030) were associated with of all-cause mortality at 1 month but not at 2 years after adjustment for potential confounders (Additional file 1: Table S1 and Table S2).

### Transaminases stratification and clinical outcome

Values for the percentages of ALT and AST in males and females in the study population are shown (Additional file 1: Table S3). The odds ratio (OR) for all-cause mortality at 2 years for participants with ALT ≥95th percentage was 5.370 (95% CI: 2.899–9.948), 7.034 (95% CI: 3.718–13.307) after adjustment for age and gender and 1.051 (95% CI: 0.302–3.652) after adjustment for all covariables. The OR for all-cause mortality at 2 years for participants with AST ≥95th percentage was 5.370 (95% CI 2.899–9.948), 5.699 (95% CI 3.030–10.718) after adjustment for age and gender and 1.796 (95% CI: 0.588–5.481) after adjustment for all covariables (Table 4). For predicting outcomes, we constructed the receiver operating characteristic area under the curve (ROC-AUC) to value the best cut-off of ALT and AST. Using a cut-off level of 63.65 IU/L, ALT predicted 1 month mortality with a sensitivity of 75.19% and specificity of 50.85% (ROC-AUC: 0.635, 95% CI: 0.552 to 0.719, *P* < 0.001) and 2 year mortality with a sensitivity of 75.56% and specificity of 44.26% (ROC-AUC: 0.604, 95% CI: 0.546 to 0.662, *P* < 0.001). Using a cut-off level of 310.5 IU/L, AST predicted 1 month mortality with a sensitivity of 84.56% and specificity of 42.37% (ROC-AUC: 0.665, 95% CI: 0.587 to 0.743, *P* < 0.001) and 2 year mortality with a sensitivity of 84.75% and specificity of 31.97% (ROC-AUC: 0.577, 95% CI: 0.520 to 0.634, *P* = 0.004).

**Table 4:** Association of liver enzymes with long-term all-cause mortality

	Association of ALT with all-cause mortality									
Category	ALT (unit/L)	Crude univariate model			Adjusted for age and sex			Adjusted for all covariables		
		OR	95% CI	*P*-value	OR	95% CI	P-value	OR	95% CI	*P*-value
ALT (1)^a^	≤23	1 (ref)		<0.001	1 (ref)		<0.001	1 (ref)		<0.001
ALT (2)	>23 to ≤39	0.957	0.534–1.714		1.126	0.624–2.030		1.358	0.650–2.834	
ALT (3)	>39 to ≤64	0.914	0.506–1.648		1.124	0.618–2.041		1.413	0.658–3.036	
ALT (4)	>64 to ≤125	1.654	0.957–2.858		2.144	1.226–3.751		1.650	0.732–3.719	
ALT (5)	≥125	5.370	2.899–9.948		7.034	3.718–13.307		1.051	0.302–3.652	
	Association of AST with all-cause mortality									
Category	AST (unit/L)	Crude univariate model			Adjusted for age and sex			Adjusted for all covariables		
		OR	95% CI	*P*-value	OR	95% CI	*P*-value	OR	95% CI	*P*-value
AST (1)^a^	≤32	1 (ref)		<0.001	1 (ref)		<0.001	1 (ref)		<0.001
AST (2)	>32 to ≤93	1.442	0.844–2.462		1.432	0.834–2.459		1.379	0.700–2.717	
AST (3)	>93 to ≤233	0.785	0.425–1.448		0.744	0.401–1.382		0.786	0.361–1.715	
AST (4)	>233 to ≤492	1.206	0.672–2.164		1.152	0.639–2.080		0.989	0.439–2.225	
AST (5)	≥492	5.370	2.899–9.948		5.699	3.030–10.718		1.796	0.588–5.481	

*HR* hazard ratio, *CI* confidence interval, *ALT* alanine aminotransferase, *AST* aspartate aminotransferase

^a^Percentiles are shown in categories: (1) ≤25th percentage; (2) 25th - < 50th percentage; (3) 50th- < 75th percentage; (4) 75th- < 95th percentage; (5) ≥95th percentage

**Fig. 2:**
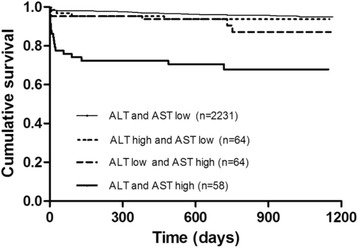
Kaplan-Meier analysis. All-cause mortality according to combinations generated by having low or high values for ALT and AST. ALT and AST were defined low if under 95th percentage, high if above 95th percentage. ALT 95th percentage value = 125 unit/L, AST 95th percentage value = 492 unit/L

The 2 year all-cause mortality was significantly high along with an increasing level of ALT and AST (Table 4). Kaplan-Meier curves were constructed to explore the additive prognostic value of combinations of ALT and AST levels. We divided the study patients into four groups by ≥95th percentage of ALT and AST or not. The all-cause mortality at 1 month and 2 years were significantly increased in patients with both AST and ALT levels ≥95th percentage (*P* < 0.001) (Fig. 2).

## Discussion

Liver function tests usually comprise ALT, AST, ALP, GGT, other nonenzymatic proteins (e.g., albumin), and heme metabolites, such as bilirubin. Among these markers, AST and ALT are often elevated in patients with STEMI [17]. Serum ALT is predominantly found in the liver. AST is mainly derived from liver and, hence, also considered as a marker of liver function; however, a significant portion of AST is derived from the heart and other tissues. Nevertheless, it remains unclear whether ALT or AST provide any long- or short-term independent prognostic value in STEMI patients who underwent PCI and whether ALT and AST levels are associated with any other risk factors.

To our knowledge, this is the first prospective study evaluating the association between ALT and AST and short- and long-term all-cause mortality in STEMI patients who underwent PCI. We demonstrated in the Chinese Han ethnic group that AST and ALT levels on admission were significant correlated with Killip classification, pre-TIMI flow, infarct-related coronary artery, and cardiac troponin I. Serum transaminases ≥95th percentage were associated with a significantly increased incidence of short- and long-term all-cause mortality.

Recent studies investigating the relationship between ALT and AST with mortality have yielded conflicting results. A large national population-based cohort study conducted in the United States showed a lack of association between overall or cardiovascular-disease (CVD) mortality with ALT [18]. However, a similar study of a population-based cohort of Caucasian persons aged 55 years or older found AST and ALT are associated with all-cause mortality [19]. Furthermore, a recent meta-analysis study with aggregate data on over 9.24 million participants and 242,953 instances of all-cause mortality found a comparatively moderate association of AST with all-cause mortality and geographical variations in the association of ALT with all-cause mortality risk in general populations [7]. Interestingly, other studies have found elevation of common markers, including ALT and AST, in patients with heart failure and CVD with liver injury resulting from ischemia or congestion [20–23]. These results further confirm the association between ALT/AST and mortality risk. Considering that liver enzymes are routine parameters of liver function assessed in STEMI patients before PCI, we investigated the association between ALT/AST and all-cause mortality in the Chinese Han ethnic group.

Notably, increased ALT levels are found in STEMI patients with acute liver injury [24–26]. Cardiac disorders contribute to the liver injury [2, 4, 27, 28]. We excluded patients with chronic hepatitis to exclude liver injury derived from hepatic disorders. The infarct-related artery only strategy for patients with multivessel arteries disease presenting with STEMI in our center minimize potential impact of different strategies of complete vs. culprit-only revascularization [29]. The primary infarct-related coronary artery in patients with ALT ≥ 95th percentage was LAD. Our findings support LAD occlusion as the factor most closely associated with left ventricular ejection fraction and with measures of left ventricular regional hypofunction, especially caused by proximal LAD [30, 31].

The liver has high metabolic activity and perfusion rate, and acute circulatory changes, such as cardiogenic shock resulting from an acute myocardial infarction, directly influence hepatic blood flow [32–34]. Every 10 mmHg drop in arterial pressure decreases the hepatic blood flow by approximately 10% [33, 35]. Circulatory failure triggers compensatory mechanisms in the liver by increasing oxygen extraction from the blood up to 95%, which results in hepatocellular dysfunction and elevation of AST/ALT [36]. The assessment for the presence and severity of circulatory failure were assessed by the Killip classification through physical examination, which showed the serum level of ALT and AST were high along with the increasing of the grade of Killip classification. Because hypoxic injury to the liver is a reversible subclinical condition accounting for over 50% of dramatic serum aminotransferase activity identified in hospital admissions [37], our results demonstrating the predictive value of ALT and AST for short-term, but not long-term, all-cause mortality were not surprising. The influences of genetics on the ethnic and environmental factors, the dosage of prescription drugs, adherence to therapy and cardiac rehabilitation all had important roles on the long-term overall cardiovascular risk. Unless for ALT and AST levels ≥95th percentage, the all-cause mortality at 1 month and 2 years post-procedure both significantly increased.

### Study limitations

This study had several limitations. This was an observational study based on data from a single center and the population belonging to a single ethnic group which located in an agricultural province of Northeast China with 52% of participants living in the countryside. The crude death rate of coronary heart disease is significantly different form urban citizens and rural residents in China [38]. The proportion of rural population may influence the result and the data may not reflect the general population of STEMI patients; however, compared to a multicenter study, this study design could be an advantage. The patients’ data were imputed electronically by a relatively constant group of attending physicians. The severity and location of coronary lesions were based on visual assessments by the same operators. Ticagrelor was not available during the study period in our center which avoided different P2Y12 inhibitors affecting AST and ALT levels. The overall strategic management of patients, including PCI technique and device used during the procedure, were more homogeneous than would be in a multi-centered study. Second, we performed only a single measurement of ALT and AST for most patients admitted to our center. We tried to exclude patients with conditions potentially associated with liver cell damage, and we also excluded 5% of patients who did not have complete evaluation of liver enzymes at admission. Those limitations are balanced by our continuous admission and, in particular, by the avoidance of ascertainment bias that occurs in clinical studies of selected patients. Third, the results of our multivariate analysis may be biased due to the potential impact of important factors that are not available in our database. Fourth, validation of the cause of death was reported only in a portion of our cohort. Due to culture and social reasons, some patients’ families did not know the exact cause of death.

## Conclusions

In summary, to our knowledge, this is the first study to investigate the value of serum transaminases in patients with STEMI treated with primary PCI. Elevated transaminases significant correlated with Killip classification, pre-TIMI flow, infarct-related coronary artery, and cardiac troponin I. Moreover, the all-cause mortality at short- and long-term post-procedure was significantly increased in patients with levels of AST and ALT ≥95th percentage.

## Additional file

Additional file 1: Three additional tables. **Table S1.** Predictors of major events at 1 month. **Table S2.** Predictors of major events at 24 month. **Table S3.** Percentile values of serum ALT and AST levels for men and women in the study population. (DOC 104 kb)

## Abbreviations

ALP: Alkaline phosphatase
ALT: Alanine transaminase
AST: Aspartate aminotransferase
CKMB: Creatine kinase MB
GGT: ɤ-glutamyl transpeptidase
HDL-C: High-density lipoprotein cholesterol
LAD: Left anterior descending artery
LCX: Left circumflex coronary artery
LDL-C: Low-density lipoprotein cholesterol
NT-proBNP: N-terminal pro-brain natriuretic peptide
PCI: Percutaneous coronary intervention
RCA: Right coronary artery
ROC-AUC: Receiver operating characteristic area under the curve
STEMI: ST-segment elevation myocardial infarction
TC: Total cholesterol
TG: Triglyceride
TIMI: Thrombolysis-in-myocardial-infarction
Tn I: Cardiac troponin I
