# Supplementary material for: Association of serum transaminases with short- and long-term outcomes in patients with ST-elevation myocardial infarction undergoing primary percutaneous coronary intervention
Source: BMC Cardiovasc Disord. 2017 Jan 28;17:43. doi: 10.1186/s12872-017-0485-6 (PMC5273829; doi:10.1186/s12872-017-0485-6)
Supplement: Additional file 1: — Three additional tables. Table S1. Predictors of major events at 1 month. Table S2. Predictors of major events at 24 month. Table S3. Percentile values of serum ALT and AST levels for men and women in the study population. (DOC 104 kb) [file 12872_2017_485_MOESM1_ESM.doc]

**Additional file 1**

**Table S1. Predictors of major events at 1 month**

| **Category** | **Model 1** | | | **Model 2** | | |
| --- | --- | --- | --- | --- | --- | --- |
|  | **HR** | **95% CI** | **P-value** | **HR** | **95% CI** | **P-value** |
| Age (year) | 1.041 | 1.010-1.072 | 0.008 | 1.045 | 1.019-1.073 | 0.001 |
| Male, n (%) | 1.167 | 0.547-2.493 | 0.689 |  |  |  |
| Living in the city | 1.590 | 0.800-3.159 | 0.186 |  |  |  |
| Hospital days | 0.803 | 0.719-0.897 | <0.001 | 0.785 | 0.705-0.875 | <0.001 |
| Dyslipidemia (n) | 0.841 | 0.425-1.664 | 0.618 |  |  |  |
| Diabetes mellitus (n) | 1.348 | 0.601-3.023 | 0.468 |  |  |  |
| Hypertension (n) | 1.170 | 0.610-1.664 | 0.618 |  |  |  |
| Myocardial disease (n) | 1.558 | 0.596-4.073 | 0.366 |  |  |  |
| Killip classification | 2.818 | 2.180-3.642 | <0.001 | 2.481 | 2.006-3.068 | <0.001 |
| Creatinine (mmol/L) | 0.998 | 0.990-1.007 | 0.701 |  |  |  |
| Glucose (mmol/L) | 1.087 | 1.009-1.170 | 0.028 | 1.082 | 1.030-1.136 | 0.002 |
| ALT (unit/L) | 1.004 | 1.001-1.006 | 0.010 | 1.002 | 1.001-1.004 | 0.001 |
| AST (unit/L) | 0.999 | 0.998-1.000 | 0.030 | 0.999 | 0.999-1.000 | 0.010 |
| alkaline phosphatase (unit/L) | 1.002 | 0.986-1.019 | 0.806 |  |  |  |
| ɤ-glutamyl transpeptidase(unit/L) | 0.997 | 0.989-1.005 | 0.467 |  |  |  |
| Cardiac troponin I (ng/mL) | 1.002 | 0.997-1.007 | 0.385 |  |  |  |
| Creatine kinase MB (ng/mL) | 1.001 | 0.997-1.006 | 0.652 |  |  |  |
| NT-proBNP (pg/mL) | 1.000 | 0.999-1.000 | 0.315 |  |  |  |
| MYO (ng/mL) | 1.000 | 0.999-1.000 | 0.993 |  |  |  |

HR, hazard ratio; CI, confidential interval; STEMI, ST-elevation myocardial infarction; HDL-C, high-density lipoprotein cholesterol; LDL-C, low-density lipoprotein cholesterol; ALT, alanine aminotransferase; AST, aspartate aminotransferase; NT-proBNP, N-terminal pro-brain natriuretic peptide; MYO, myoglobin.

**Table S2. Predictors of major events at 24 month**

| **Category** | **Model 1** | | | **Model 2** | | |
| --- | --- | --- | --- | --- | --- | --- |
|  | **HR** | **95% CI** | **P-value** | **HR** | **95% CI** | **P-value** |
| Age (year) | 1.038 | 1.017-1.058 | <0.001 | 1.044 | 1.027-1.061 | <0.001 |
| Male, n (%) | 0.825 | 0.515-1.322 | 0.424 |  |  |  |
| Living in the city | 1.375 | 0.887-2.132 | 0.154 |  |  |  |
| Hospital days | 0.974 | 0.930-1.020 | 0.265 |  |  |  |
| Dyslipidemia (n) | 0.688 | 0.436-1.086 | 0.109 |  |  |  |
| Diabetes mellitus (n) | 0.891 | 0.508-1.564 | 0.688 |  |  |  |
| Hypertension (n) | 1.121 | 0.739-1.701 | 0.592 |  |  |  |
| Myocardial disease (n) | 1.984 | 1.114-3.532 | 0.020 | 1.716 | 1.024-2.876 | 0.040 |
| Killip classification | 2.011 | 1.687-2.396 | <0.001 | 2.125 | 1.838-2.456 | <0.001 |
| Creatinine (mmol/L) | 1.000 | 0.994-1.006 | 0.948 |  |  |  |
| Glucose (mmol/L) | 1.027 | 0.967-1.090 | 0.388 |  |  |  |
| ALT (unit/L) | 1.002 | 1.000-1.004 | 0.056 |  |  |  |
| AST (unit/L) | 1.000 | 0.999-1.000 | 0.381 |  |  |  |
| alkaline phosphatase (unit/L) | 1.001 | 0.991-1.011 | 0.892 |  |  |  |
| ɤ-glutamyl transpeptidase (unit/L) | 1.001 | 0.998-1.003 | 0.585 |  |  |  |
| Cardiac troponin I (ng/mL) | 1.001 | 0.998-1.004 | 0.475 |  |  |  |
| Creatine kinase MB (ng/ml) | 0.999 | 0.995-1.002 | 0.476 |  |  |  |
| NT-proBNP (pg/mL) | 1.000 | 1.000-1.000 | 0.169 |  |  |  |
| MYO (ng/ml) | 1.000 | 0.999-1.000 | 0.503 |  |  |  |

HR, hazard ratio; CI, confidence interval; STEMI, ST-elevation myocardial infarction; HDL-C, high-density lipoprotein cholesterol; LDL-C, low-density lipoprotein cholesterol; ALT, alanine aminotransferase; AST, aspartate aminotransferase; NT-proBNP, N-terminal pro-brain natriuretic peptide; MYO, myoglobin.

**Table S3. Percentile values of serum ALT and AST levels for men and women in the study population**

|  | <25th percentage | 25th-<50th percentage | 50th-<75th percentage | 75th-<95th percentage | ≥95th percentage |
| --- | --- | --- | --- | --- | --- |
| ALT **(unit/L)** | ≤23 | >23 to ≤39 | >39 to ≤64 | >64 to ≤125 | ≥125 |
| Male | ≤25 | >25 to ≤42 | >42 to ≤67 | >67 to ≤125 | ≥125 |
| Female | ≤19 | >19 to ≤33 | >33 to ≤56 | >56 to ≤121 | ≥121 |
| AST **(unit/L)** | ≤32 | >32 to ≤93 | >93 to ≤233 | >233 to ≤492 | ≥492 |
| Male | ≤31 | >31 to ≤91 | >91 to ≤244 | >244 to ≤501 | ≥501 |
| Female | ≤34 | >34 to ≤99 | >99 to ≤205 | >205 to ≤413 | ≥413 |

ALT, alanine aminotransferase; AST, aspartate aminotransferase.
